# Association of thrombotic microangiopathy with interferon therapy for hepatitis B: a case report

**DOI:** 10.1186/s13256-024-04635-8

**Published:** 2024-07-05

**Authors:** Shan Wei, Wenjuan Mei, Ying Wang

**Affiliations:** 1 Nephrology Department, The First Affiliated Hospital, Jiangxi Medical College, Nanchang University, Nanchang, 330006 China; 2 Nanchang University, Nanchang, 330031 China

**Keywords:** Thrombotic microangiopathy (TMA), Interferon, Renal injury

## Abstract

**Background:**

Thrombotic microangiopathy is characterized by microangiopathic hemolytic anemia, thrombocytopenia, and organ injury. The pathological features include vascular damage that is manifested by arteriolar and capillary thrombosis with characteristic abnormalities in the endothelium and vessel wall. Thrombocytopenia is one of the common adverse effects of interferon therapy. However, a more serious but rare side effect is thrombotic microangiopathy.

**Case presentation:**

We report the case of a 36-year-old Asian male patient with clinical manifestations of hypertension, blurred vision, acute renal failure, thrombocytopenia, and thrombotic microangiopathy. Renal biopsy showed interstitial edema with fibrosis, arteriolar thickening with vitreous changes, and epithelial podocytes segmental fusion. Immunofluorescence microscopy showed C3(+), Ig A(+) deposition in the mesangial region, which was pathologically consistent with thrombotic microangiopathy renal injury and Ig A deposition. The patient had a history of hepatitis B virus infection for more than 5 years. Lamivudine was used in the past, but the injection of long-acting interferon combined with tenofovir alafenamide fumarate was used since 2018. The comprehensive clinical investigation and laboratory examination diagnosed the condition as thrombotic microangiopathy kidney injury caused by interferon. After stopping interferon in his treatment, the patient’s renal function partially recovered after three consecutive therapeutic plasma exchange treatments and follow-up treatment without immunosuppressant. The renal function of the patient remained stable.

**Conclusions:**

This report indicates that interferon can induce thrombotic microangiopathy with acute renal injury, which can progress to chronic renal insufficiency.

## Background

Thrombotic microangiopathy (TMA) is a condition that affects multiple systems and causes ischemic symptoms in the brain and kidneys due to platelet aggregation in arterial microcirculation. The root causes of TMA are abnormal platelet aggregation and endothelial cell injury [1]. TMA can be primary or secondary. The primary type of TMA includes classical hemolytic uremic syndrome, while the secondary type is caused by different factors such as systemic diseases, drugs, or pregnancy. TMA patients often have a history of taking medications, and several drugs are believed to be responsible for causing TMA, such as cyclosporine, ticlopidine, clopidogrel, and quinine. Additionally, interferon (IFN) has been linked to TMA, including thrombotic thrombocytopenic purpura (TTP) and hemolytic uremic syndrome (HUS). Interferon has also been known to cause kidney damage, leading to several conditions, such as focal segmental glomerulosclerosis [2], membranous glomerulonephritis [3], acute renal failure [4], lupus nephritis [5], and thrombotic microangiopathy [6–9]. We present a case study of a patient who suffered from acute kidney injury due to the deposition of immunoglobulin A (IgA) after receiving long-term injections of interferon-alpha 2a. The renal injury was diagnosed as TMA, and this case highlights the potential adverse effects of this medication on kidney function.

## Case presentation

**Fig. 1:**
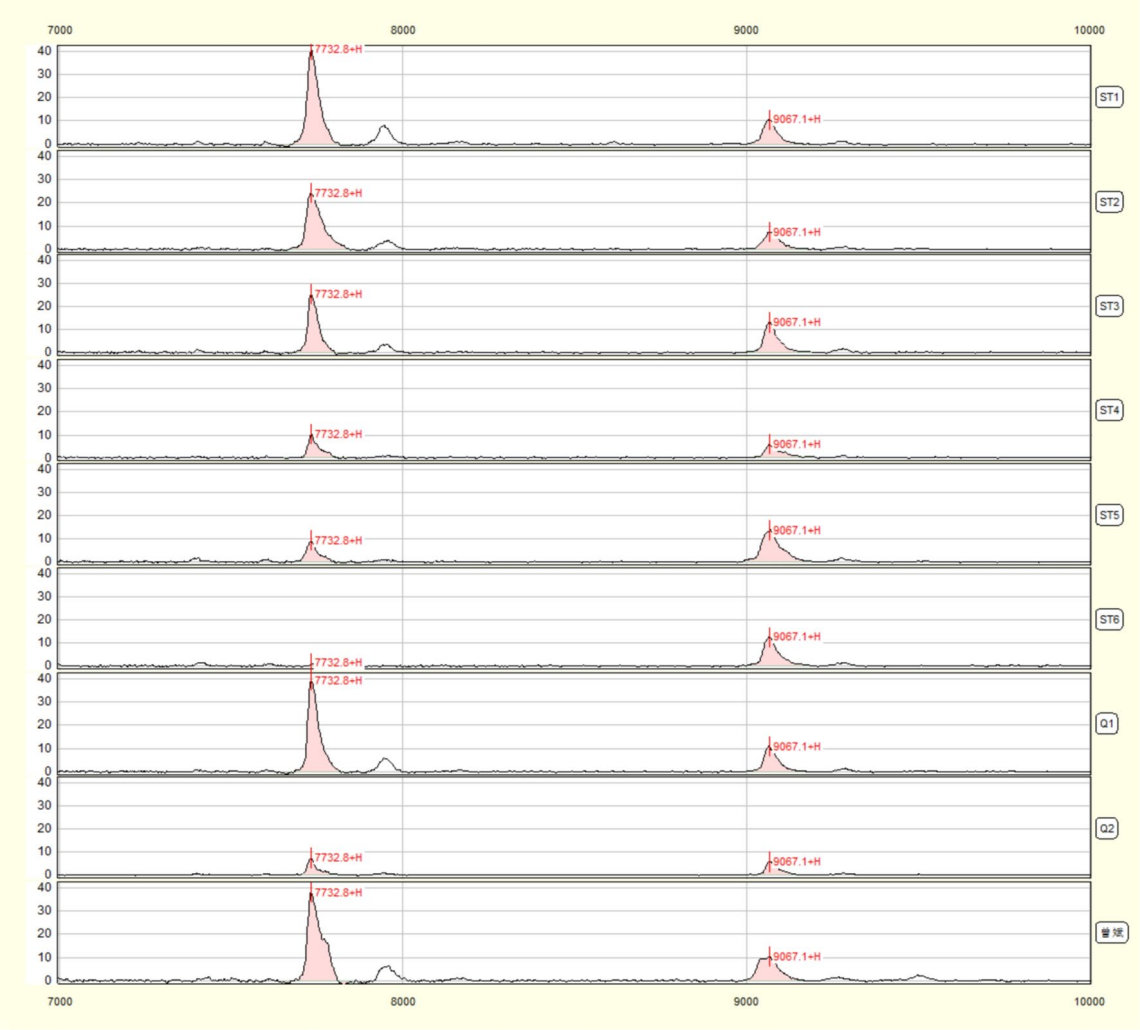
Surface-enhanced laser desorption ionization time-of-flight mass spectrometry (SELDI-TOF) map of ADAMTS13 activity

The 36-year-old Asian patient presented to our hospital on 20 March 2021 because of blurred vision and dizziness. He attended the Affiliated Ophthalmology Hospital of Nanchang University with the symptoms of suddenly blurred vision 2 weeks ago, and was diagnosed as “left central serous retinal chorioidea changes.” He was then treated with traditional Chinese medicine. However, his blurred vision abruptly aggravated 1 day prior to the intended treatment, so he was referred to the ophthalmology department of our hospital where the patient’s blood pressure up to 184/133 mmHg was noticed. The patient was then reassigned to the cardiovascular department; his other laboratory results were discovered, including urine protein 3+, erythrocyte 108 cells/μl, and creatinine 253 μmol/L. The patient was transferred to our department, that is, the department of nephrology. The patient had a history of hepatitis B virus (HBV) infection for more than 5 years, and was initially treated with lamivudine and later changed to injectable long-acting interferon once per week and oral tenofovir alafenamide fumarate (TAF) since 2018. Physical examination showed a chronic facial features without eyelid or bilateral lower extremity edema, and no other obvious abnormalities. The results of laboratory examination were as follows (Table 1): RBC 2.31 × 10^12^/L, Hb 75 g/L, Plt 22 × 10^9^/L, ESR 24 mm/hour, Tbil 22.5 μmol/L, Scr 239 μmol/L, Bun 8.9 mmol/L, UA 401.4 μmol/L, LDH 462.3 U/L, IgA 4.85 g/L, IgM 0.34 g/L, C3 0.86 g/L, C4 0.25 g/L, HBsAg > 250.00 IU/ml (+), HBeAg 0.992 PEI U/ml (+), HBcAb 8.518S/CO (+), FT3 3.19 pg/ml, FT4 1.4 ng/dl, sTSH 9.55 μIU/ml, 24H urine protein 1.58 g/24 hour, P/CR 0.57, and the negative outcomes for dsDNA, ANA, ANCA, anti-GBM, Ham’s test, and Coomb’s test. Broken erythrocytes were occasionally seen in peripheral blood. Other conditions included: ALD 119.9 ng/dl, AngI 2.49 ng/dl, PRA 8.856 ng/ml/hour, AngII 169.5 pg/ml, IL-5/26.4 pg/ml, IL-2 9.23 pg/ml, IFN-α 29.04 pg/ml, IL-6 24.67 pg/ml, IL-1β 226.53 pg/ml, INF-r 65.14 pg/ml, IL-8 198.49 pg/ml, TNF-α 25.19 pg/ml, left kidney GFR: 14.22 ml/minute, right kidney GFR: 14.79 ml/minute, and total kidney GFR: 29.01 ml/minute. Color doppler ultrasonography showed that reductions in both kidney volume (left 8.8 × 3.9 × 5 × 4.4 cm, right 9.2 × 5.0 × 4.1 cm), the thickness of cortex being approximately 0.6 cm, and a decrease in the blood flow of kidneys. Computed tomography (CT) scan of lungs showed mild emphysema at the top and mild chronic infections. Fundus photography indicated bleeding and exudation in both eyes. Thrombocytopenia, elevated lactic dehydrogenase (LDH), and acute renal failure are the common elements of all TMAs. To corroborate our speculation, we immediately performed an ADAMTS13 activity determination, with a result of 58.6% (Fig. 1).

**Table 1:** Patient’s laboratory values during hospital admission

	One^a^	Five^a^	10st^a^	Three month^a^	Six month^a^	One year^a^
ALT (9–50 U/L)	16.6	11	20.9	12.1	17.9	18.7
AST (15–40 U/L)	54	14.7	19.2	18.9	14.3	21.2
Alb (40–55 g/L)	44.8	40.3	42.1	46.1	40.9	48.4
Tbil (3.42–20.5 μmol/L)	22.5	6.3	2.9	1.2	2.2	1.7
Scr (57–97 μmol/L)	239	259.3	238.6	179.2	126.1	143.3
Bun (3.1–8.0 mmol/L)	8.9	12.1	8.9	6.9	3	7.2
UA (208–428 μmol/L)	413.7	394.2	405.4	474.8	360	461.9
LDH (120–250 U/L)	165.1	462.3	165.1	–	198.9	–
Hb (130–175 g/L)	75	72	87	112	117	131
RBC (4.0–5.5 × 10^12^/L)	2.31	2.28	2.76	3.68	3.95	4.3
PLT (125–350 × 10^9^/L)	22	86	231	178	254	148
ESR (0–20 mm/hour)	24	–	20	–	–	–
Proteinuria (0–0.15 g/24 hour)	1.58	0.57	0.14	0.1	0.03	0.06

*ALT* alanine aminotransferase, *AST* aspartate transaminase, *Alb* albumin, *Tbil* total bilirubin, *Scr* serum creatinine, *Bun* blood urea nitrogen, *UA* uric acid, *LDH* Lactic dehydrogenase, *Hb* hemoglobin, *RBC* red blood cell, *Plt* platelet, *ESR* erythrocyte sedimentation rate, *HBsAg* Hepatits B surface antigen, *HBeAg* Hepatitis B e antigen, *HBcAb* Hepatitis B core antigen, *FT3*, Free triiodothyronine; *FT4* Free thyroxine, *sTSH* thyroid stimulating hormone, *ALD* aldosterone, *AngII* Angiotensin, *PRA* Plasma renin activity, *GFR* Glomerular filtration rate

^a^Days post admission

**Fig. 2:**
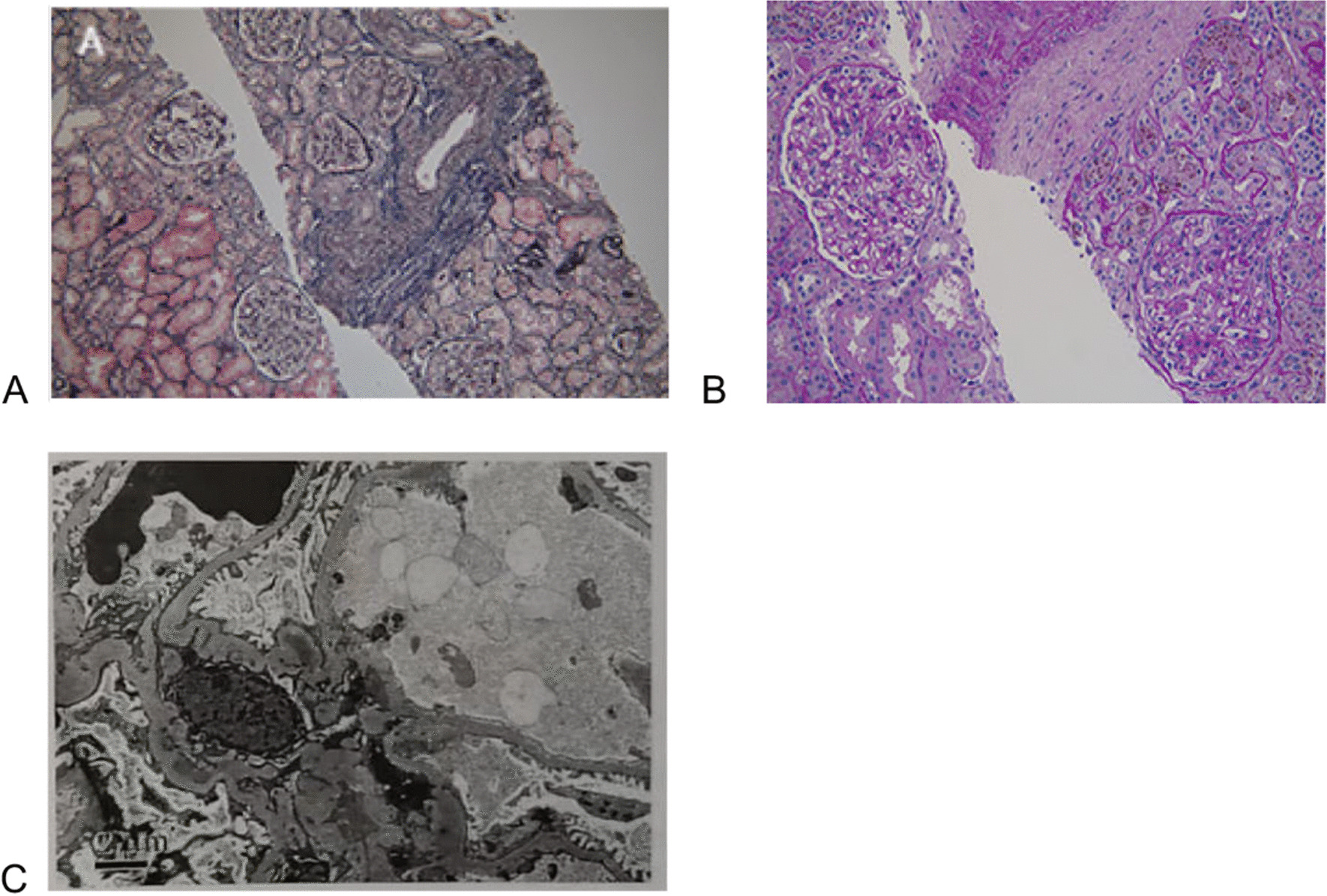
Renal pathology of our patient at diagnosis. **A** PASM + Masson staining ×100 showed glomerular ischemia and shrinkage, segmental proliferation of mesangial cells and matrix. **B** PAS staining ×200 revealed glomerular ischemic sclerosis and lymphocyte and monocyte infiltration with fibrosis. **C** Electron microscope discovered the segments of the loose layer of the basement membrane widened and fusion of podocytic process

However, the patient’s dizziness and other symptoms were improved, and his blood pressure decreased to 150/90 mmHg after three times of plasma exchange (1500 ml plasma each time) every other day from 25 March. His blood pressure was treated with urapidil, metoprolol succinate, nifedipine, terazosin, benazepril, and plasma transfusion, but the renal function was not significantly recovered. To determine the cause of acute renal failure, we performed a renal biopsy, which showed glomerular ischemia and shrinkage, segmental proliferation of mesangial cells and matrix, lymphocyte infiltration, fibrosis in the renal interstitial, thickening, and hyaline change of artery with onion skin lesion. The immunofluorescence microscope showed C3 (+), IgG (−), IgA (+), IgM (+), C1q (−), FRA (−), Alb (−), κ (−), λ (−), and mesangial granular deposition under light microscope (Fig. 2A, B). The electron microscope showed that the segments of the loose layer of the basement membrane had widened and a fusion of podocytic process (Fig. 2C). Pathology revealed TMA and IgA deposition.

To exclude the possibility of genetic variants, we also performed whole-exome sequencing without any gene variations correlated to the clinical feature of this patient. Considering the patient’s past history of hepatitis B and tracing the patient’s previous blood work and blood biochemistry, ee found the PLT was 82 × 10^9^/L in January 2021 when he came to the infection department to treat the HBV in our hospital because of the history of hepatitis B. No family history of the same disease was observed. The platelet increased to 231 × 10^9^/L and 24 hour urinary protein decreased to 0.14 g after therapeutic plasma exchange, but creatinine remained at 240 µmol/L. We followed-up the patient for more than 2 years. At present, urinary protein turned negative, and the platelet was normal without special treatment. The latest examination showed the creatinine was 120.3 µmol/L in February 2023, indicating that the renal function and platelet were fully recovered after the therapeutic plasma exchange and blood pressure control.

## Discussion

We report a case of hypertension, renal insufficiency, and thrombocytopenia. Unexplained hypertension, thrombocytopenia, and microangiopathic hemolytic anemia (MAHA) indicated the clinical presentations being attributable to TMA [10]. The patient did not have a history of hypertension but suddenly presented symptoms of malignant hypertension; the situation led us to examine renal insufficiency as the underlying cause for malignant hypertension or TMA. We have taken initial comprehensive care for this patient, including termination of interferon usage, exclusion of potential cause for secondary hypertension via analyses of urological ultrasound, Aldosterone, and AngII, as well as performing ADAMTS13 activity testing, immunological marker identification, and renal biopsy. We then followed with targeted therapy consisting of TPE and blood pressure reduction, which resulted in an increase of platelet numbers to the normal level, a reduction of blood pressure to the normal range, and stabilization of renal function. Collectively, our study revealed TMA being the underlying cause for the renal pathology and all clinical presentations of the patient.

We learned that the patient had a history of hepatitis B and was treated with long-acting interferon injected once a week in combination with TAF once a day since 2018. It was reported that interferon can cause TMA with the average duration of treatment for TMA occurrence being 40.4 months [11]. Interferons (IFNs) are a family of natural cytokines that interfere with viral replication, cell proliferation, and immune regulation. There are two major types of interferons: Type 1 (IFNα produced by leukocytes and IFNβ produced by fibroblasts and epithelial cells) and type 2 [IFN γ produced by activated T cells and NK cells]. Clinical applications of exogenous IFN are well established in the therapy for cancers, hepatitis C, idiopathic pulmonary fibrosis, and multiple sclerosis [12]. IFN is associated with many adverse effects such as flu-like symptoms, asthenia, anorexia, and injection site reaction, and can lead to thyroid abnormalities in up to 30% of patients, and exacerbation of autoimmune disease [13].

Nonetheless, the mechanisms for interferon to induce TMA remain unclear. Disruption of complement regulation play a role in endothelial damage, which is characteristic of TMA [14]. IFNα has been shown to increase leukocyte adhesion to endothelial cells. This triggers endothelial damage and the subsequent release of large multimers of von Willebrand factor causing endothelial swelling, platelet aggregation, and intraluminal microthrombi formation. Activated leukocytes and their products such as TNF, IFN, IL-1, and free radicals are implicated in tissue injury [15]. The pathogenesis of TMA may also involve inhibition of vascular endothelial growth factor (VEGF) production in renal podocytes [16]. Under physiological conditions, VEGF stimulates signal transduction pathways and transcription through activation of its receptor VEGFR2 [17]. These events are essential for angiogenesis. INF-induced immune responses play a key role in the production of cross-reactive anti-ADAMTS13 antibodies and lead to microvascular pathological hemolysis; glomerular endothelial cells express and secret ADAMTS13. The low activity of ADAMTS13 is associated with the presence of an anti-ADAMTS13 IgG antibody during treatment with interferon-alpha 2a, contributing to the observed adverse side effects. Of note, ADAMTS13 is now an integral part of the initial diagnostic workup of any newly identified TMA [18]. Quantitative measurement of ADAMTS13 activity along with antigen and inhibitor levels accurately permit or rule out a diagnosis of TTP. As early recognition of TTP and prompt therapeutic intervention are critical to patient outcomes, blood for ADAMTS13 testing should be drawn prior to the first plasma exchange treatment, but diagnosis for TTP can still occur after plasma exchange therapy has been initiated [19, 20]. We have measured plasma ADAMTS 13 activity in patients immediately after admission prior to plasma replacement therapy. There is a significant early mortality risk associated with TMA induced by TTP, which can be reduced by the timely use of plasma exchange [21], and so definitive treatment should not be delayed awaiting the results of ADAMTS13 activity testing when a TMA is present and TTP is suspected.

Plasma exchange (PE) is the quickest and most effective means of removing pathogenic ADAMTS13 autoantibodies. As delays in commencement of PE are associated with increased mortality, all adults presenting with TMA should receive PE urgently as empiric therapy, until TTP has been excluded [22]. PE should be continued daily until at least 2 days after the platelet count has normalized (> 150 × 10^9^/L), bearing in mind other potential causes for any persisting thrombocytopenia [23, 24]. High-dose steroids should be used in conjunction with PE for acquired TTP, and in all patients treated empirically with PE pending the results of ADAMTS13 testing (for example, oral prednisolone 1 mg/kg per day or, for the most severe cases, pulse intravenous methylprednisolone 1 g daily for the first 3 days). Rituximab (off-label) may be appropriate once acquired TTP; initial treatment with rituximab reduces relapse rates based on prospective studies [17], and is recommended for patients presenting with poor prognostic features including neurological or cardiac involvement, and for relapsing disease [25]. Steroids and rituximab are not beneficial in congenital TTP. Recent data on TMA pathogenesis shows a common pathway of complement activation in all TMA patients [26]. In addition, eculizumab, an anti-C5 monoclonal antibody, could be effective in controlling TMA, but it remains unclear whether this monoclonal antibody would also be effective in drug-induced thrombotic microangiopathy (DITMA) until now [27].

Typically, proper management of DITMA mainly involves withdrawal of the suspected causative drug and supportive care. Resolution or improvement of TMA is observed after the drug is stopped or the dose is reduced. However, discontinuation alone is often not enough to lead to clinical recovery and some degree of kidney injury can persist. In these patients, especially in case of advanced kidney disease, other therapies need to be considered. Therapeutic plasma exchange and rituximab (RTX) have been utilized among patients not responding to causative drug withdrawal [28, 29]. In our case reported here, the patient was diagnosed with suspected interferon-induced TMA in a timely manner following his hospitalization. He was immediately discontinued from the interferon-based therapy, and given plasma exchange without waiting for the results of the renal biopsy. After the third plasma exchange, LDH and PLT returned to normal, so the patient was not treated with glucocorticoids and immunosuppressants. The patient’s renal function is continuing to recover in our follow-up treatment.

## Conclusions

This case report highlights the importance of renal biopsy and ADAMTS13 testing for a definitive diagnosis of renal injury caused by TMA, and how TMA should be treated afterward. It is crucial to perform renal biopsy and ADAMTS13 testing to accurately diagnose renal injury caused by TMA. This case report emphasizes the importance of these procedures and describes the treatment approach for TMA. Patients undergoing long-term interferon treatment should receive regular monitoring of blood routine, kidney and liver function, and thyroid function to prevent adverse effects such as thrombocytopenia, renal insufficiency, and hypothyroidism. Thrombotic microangiopathy caused by interferon is a rare side effect that appears as acute or subacute kidney injury. Our experience with this case demonstrates the significance of rapid diagnosis and treatment. It is vital not to delay initiating therapeutic plasma exchange for the renal biopsy, as this is essential for the patient’s recovery and prognosis.

## Data Availability

The datasets used and analyzed during the current study are available from the corresponding author on reasonable request.

## Abbreviations

TMA: Thrombotic microangiopathy
TAF: Tenofovir alafenamide fumarate
HAMA: Hemolytic anemia
HUS: Hemolytic uremic syndrome
HBV: Hepatitis B virus
TPE: Therapeutic plasma exchange
TTP: Thrombotic thrombocytopenic purpura
MAHA: Microangiopathic hemolytic anemia
IFN: Interferons
VEGF: Vascular endothelial growth factor
RTX: Rituximab
DITMA: Drug-induced thrombotic microangiopathy

## References

[1] Ruggenenti P, Noris M, Remuzzi G (2001). Thrombotic microangiopathy, hemolytic uremic syndrome, and thrombotic thrombocytopenic purpura. Kidney Int.

[2] Markowitz GS, Nasr SH, Stokes MB, D'Agati VD (2010). Treatment with IFN-{alpha}, -{beta}, or -{gamma} is associated with collapsing focal segmental glomerulosclerosis. Clin J Am Soc Nephrol.

[3] Trimarchi H, Coppo R (2021). Glomerular endothelial activation, C4d deposits and microangiopathy in immunoglobulin A nephropathy. Nephrol Dial Transplant.

[4] Fahal IH, Murry N, Chu P, Bell GM (1993). Acute renal failure during interferon treatment. BMJ.

[5] Abbott IJ, Chang CC, Skinner MJ, Street A, Perry G, McLean C (2009). Development and management of systemic lupus erythematosus in an HIV-infected man with hepatitis C and B co-infection following interferon therapy: a case report. J Med Case Rep.

[6] Honda K, Ando A, Endo M, Shimizu K, Higashihara M, Nitta K (1997). Thrombotic microangiopathy associated with alpha-interferon therapy for chronic myelocytic leukemia. Am J Kidney Dis.

[7] Badid C, McGregor B, Faivre JM, Guerard A, Juillard L, Fouque D (2001). Renal thrombotic microangiopathy induced by interferon-alpha. Nephrol Dial Transplant.

[8] Magee CC (2001). Renal thrombotic microangiopathy induced by interferon-alpha. Nephrol Dial Transplant.

[9] Lotta LA, Degasperi E, Aghemo A, Ferrari B, Peyvandi F, Colombo M (2013). Treatment of chronic hepatitis C with pegylated interferon-α in a patient with recurrent autoimmune thrombotic thrombocytopenic purpura. Transfus Med.

[10] Fox LC, Cohney SJ, Kausman JY, Shortt J, Hughes PD, Wood EM (2018). Consensus opinion on diagnosis and management of thrombotic microangiopathy in Australia and New Zealand. Intern Med J.

[11] Kundra A, Wang JC (2017). Interferon induced thrombotic microangiopathy (TMA): analysis and concise review. Crit Rev Oncol Hematol.

[12] Walther EU, Hohlfeld R (1999). Multiple sclerosis: side effects of interferon beta therapy and their management. Neurology.

[13] Costelloe SJ, Wassef N, Schulz J, Vaghijiani T, Morris C, Whiting S (2010). Thyroid dysfunction in a UK hepatitis C population treated with interferon-alpha and ribavirin combination therapy. Clin Endocrinol (Oxf).

[14] Broughton A, Cosyns JP, Jadoul M (2011). Thrombotic microangiopathy induced by long-term interferon-β therapy for multiple sclerosis: a case report. Clin Nephrol.

[15] Galesic K, Bozic B, Racic I, Scukanec-Spoljar M (2006). Thrombotic microangiopathy associated with alpha-interferon therapy for chronic myeloid leukaemia. Nephrology (Carlton).

[16] Mahe J, Meurette A, Moreau A, Vercel C, Jolliet P (2013). Renal thrombotic microangiopathy caused by interferon beta-1a treatment for multiple sclerosis. Drug Des Devel Ther.

[17] George JN, Terrell DR, Vesely SK, Kremer Hovinga JA, Lämmle B (2012). Thrombotic microangiopathic syndromes associated with drugs, HIV infection, hematopoietic stem cell transplantation and cancer. Presse Med.

[18] Campistol JM, Arias M, Ariceta G, Blasco M, Espinosa L, Espinosa M (2015). An update for atypical haemolytic uraemic syndrome: diagnosis and treatment. A consensus document Nefrologia.

[19] Sadler JE (2015). What’s new in the diagnosis and pathophysiology of thrombotic thrombocytopenic purpura. Hematology Am Soc Hematol Educ Program.

[20] Wu N, Liu J, Yang S, Kellett ET, Cataland SR, Li H (2015). Diagnostic and prognostic values of ADAMTS13 activity measured during daily plasma exchange therapy in patients with acquired thrombotic thrombocytopenic purpura. Transfusion.

[21] Scully M, Yarranton H, Liesner R, Cavenagh J, Hunt B, Benjamin S (2008). Regional UK TTP registry: correlation with laboratory ADAMTS 13 analysis and clinical features. Br J Haematol.

[22] Clark WF (2012). Thrombotic microangiopathy: current knowledge and outcomes with plasma exchange. Semin Dial.

[23] Scully M, Hunt BJ, Benjamin S, Liesner R, Rose P, Peyvandi F (2012). Guidelines on the diagnosis and management of thrombotic thrombocytopenic purpura and other thrombotic microangiopathies. Br J Haematol.

[24] Sarode R, Bandarenko N, Brecher ME, Kiss JE, Marques MB, Szczepiorkowski ZM (2014). Thrombotic thrombocytopenic purpura: 2012 American Society for Apheresis (ASFA) consensus conference on classification, diagnosis, management, and future research. J Clin Apher.

[25] Page EE, Kremer Hovinga JA, Terrell DR, Vesely SK, George JN (2016). Rituximab reduces risk for relapse in patients with thrombotic thrombocytopenic purpura. Blood.

[26] Noris M, Mescia F, Remuzzi G (2012). STEC-HUS, atypical HUS and TTP are all diseases of complement activation. Nat Rev Nephrol.

[27] Loirat C, Fakhouri F, Ariceta G, Besbas N, Bitzan M, Bjerre A (2016). An international consensus approach to the management of atypical hemolytic uremic syndrome in children. Pediatr Nephrol.

[28] Gourley BL, Mesa H, Gupta P (2010). Rapid and complete resolution of chemotherapy-induced thrombotic thrombocytopenic purpura/hemolytic uremic syndrome (TTP/HUS) with rituximab. Cancer Chemother Pharmacol.

[29] Murugapandian S, Bijin B, Mansour I, Daheshpour S, Pillai BG, Thajudeen B (2015). Improvement in gemcitabine-induced thrombotic microangiopathy with rituximab in a patient with ovarian cancer: mechanistic considerations. Case Rep Nephrol Dial.

